# Latest Trends in Surface Modification for Dental Implantology: Innovative Developments and Analytical Applications

**DOI:** 10.3390/pharmaceutics14020455

**Published:** 2022-02-21

**Authors:** Francesca Accioni, Juan Vázquez, Manuel Merinero, Belén Begines, Ana Alcudia

**Affiliations:** 1 Departamento de Química Orgánica y Farmacéutica, Universidad de Sevilla, 41012 Seville, Spain; francesca.accioni@gmail.com (F.A.); lolo191995@gmail.com (M.M.); 2 Departamento de Química Orgánica, Universidad de Sevilla, 41012 Seville, Spain; cabello@us.es; 3 Departamento de Citología e Histología Normal y Patológica, Universidad de Sevilla, 41012 Seville, Spain

**Keywords:** dental implants, osseointegration, antimicrobial activity, inorganic coatings, polymeric coatings, nanotechnology, drug delivery, biomedical applications, pharmaceutical agents, biomaterials

## Abstract

An increase in the world population and its life expectancy, as well as the ongoing concern about our physical appearance, have elevated the relevance of dental implantology in recent decades. Engineering strategies to improve the survival rate of dental implants have been widely investigated, focusing on implant material composition, geometry (usually guided to reduce stiffness), and interface surrounding tissues. Although efforts to develop different implant surface modifications are being applied in commercial dental prostheses today, the inclusion of surface coatings has gained special interest, as they can be tailored to efficiently enhance osseointegration, as well as to reduce bacterial-related infection, minimizing peri-implantitis appearance and its associated risks. The use of biomaterials to replace teeth has highlighted the need for the development of reliable analytical methods to assess the therapeutic benefits of implants. This literature review considers the state-of-the-art strategies for surface modification or coating and analytical methodologies for increasing the survival rate for teeth restoration.

## 1. Introduction

Dental implants are most similar to natural teeth in their mastication and aesthetics; they are also biocompatible and require biocompatibility, masticatory feature, and aesthetic follow-up [1,2,3]. The American Association of Oral and Maxillofacial Surgeons estimated that two million implants are placed per year worldwide. The longevity of the population and the demand for cosmetic dentistry have led to their increasing use [2,4].

**Figure 1:**
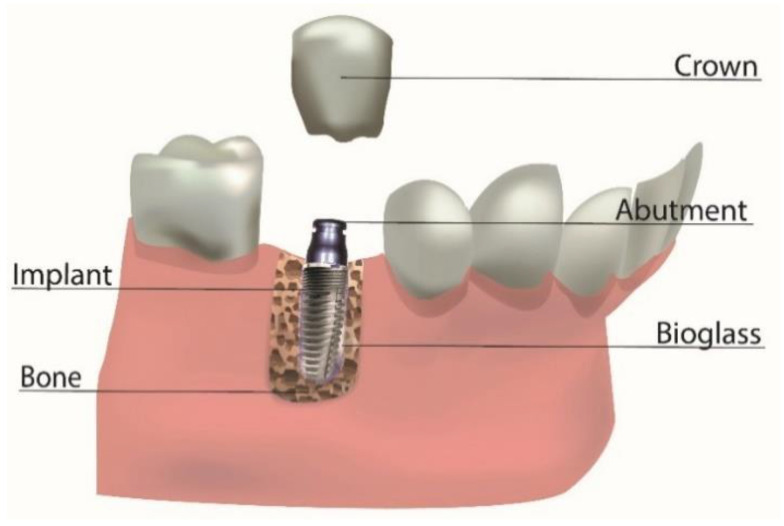
Modern implant placed in oral cavity.

Implants are expected to have a 90% success rate after 10 to 15 years of implantation. However, between 5% and 11% of dental implants do not result in the required osseointegration in the maxillofacial bone. A startling phenomenon that has arisen from the widespread use of dental implants is the health issue related to peri-implant disease [5]. The failure of the long-term stability of the dental implant occurs because of biological (20% because of peri-implantitis, from microbial plaque or bacterial infections [6,7]), and mechanical causes, (stress shielding causing osteopenia and clenching–bruxism overloading interfacial bone [8,9,10,11]). Figure 1 shows a schematic representation of a modern implant.

**Figure 2:**
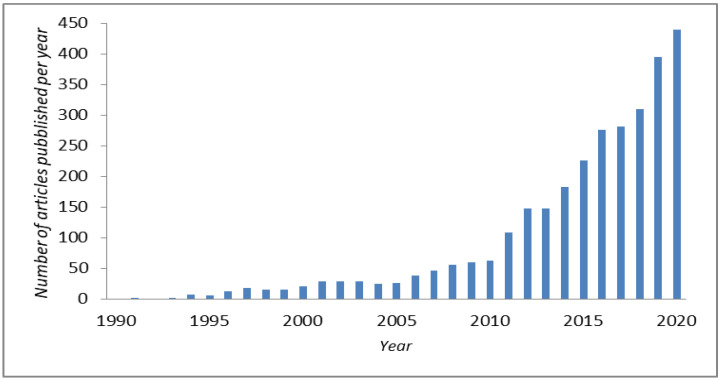
Trend of dental implants research according to the Web of Science database.

The goal of researchers has been to replace normal teeth function and prevent the peri-implantitis issues using dental implants made of novel materials that trigger the osseointegration processes. Figure 2 shows a clear increase implant research [2] from the Web of Science database. Data were obtained using filters with the keyword “peri-implantitis”, refined by review articles with the timespan 1990–2020. 

Osteointegration is triggered by a cascade mechanism starting with interfacial reactions between the implant surface and the blood cells and connective tissue [12,13]. Bone trauma to place the implants creates a fibronectin rich blood clot pillar for the cells to form a new tissue [14]. Subsequently, osteogenic cells start to release mineralized collagenous substance between the implant and the host. Eventually, bone remodeling triggers new bone formation [15].

Peri-implantitis has been described in the World Workshop on the Classification of Periodontal and Peri-implant Diseases and Conditions as “a plaque-associated pathological condition occurring in tissues around dental implants, characterized by inflammation in the peri-implant mucosa and subsequent progressive loss of supporting bone” [16,17], which can results in treatment failure [18]. Peri-implantitis can lead to an irreversible, infectious illness generated by multi-factorial risks that are grouped into the following four categories: excessive mechanical solicitations, bruise of peri-implant tissue, corrosion and colonization of pathogenic microorganisms [19].

In this sense, five hundred bacteria live in symbiosis in the oral cavity and can colonize the implant when plankton bacteria adhere to the biomaterial by van der Waals or gravitational forces. Their flagella, pili and proteins form small aggregates of bacteria that secret polysaccharides and proteins for the formation of a biofilm. Biofilms plays a key role in the protection of bacteria colonization from immune system cells and antibiotics (e.g., antibiotics resistance) [20,21,22,23,24,25]. Moreover, bacteria can induce apoptosis, and/or activate a cascade of proinflammatory molecules, boosting the osteoclastogenesis process to bone resorption. Bacterial colonization and biofilm formation enhance the risk of implant loss via peri-implantitis [26,27,28,29,30,31].

**Figure 3:**
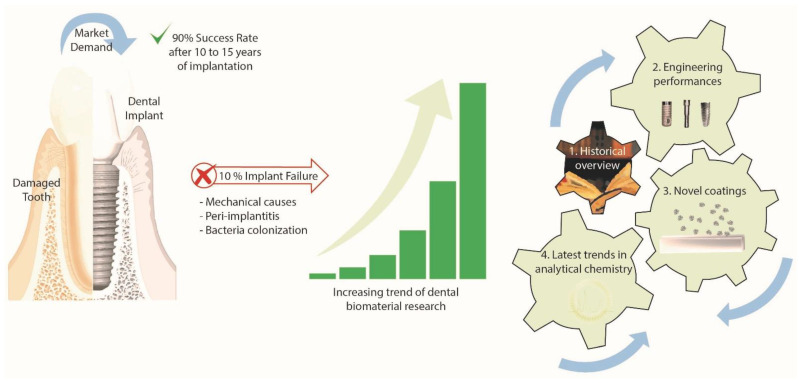
Graphical overview of the review.

This review includes a brief historical overview followed by 1-engineering strategies in dental implants; 2-coatings; 3-trends in analytical chemistry to cope with both osseointegration with the host bone tissue, and peri-implantitis issues. A graphical overview is presented in Figure 3. 

The latest engineering performances regarding dental implants are discussed. The topographic and/or physicochemical modification of implant surfaces leads to the capacity for osseointegration. The second part on novel coatings is based on biomaterials with osteoconductive and/or antimicrobial activities such as killing organisms that adhere to the implant or reduce the microbes’ adherence because of its antifouling activity or release of drugs. The third part describes the latest trends in analytical chemistry that have supported dental implantology research to facilitate future methodological studies.

## 2. Historical Overview of Implantology

**Scheme 1:**
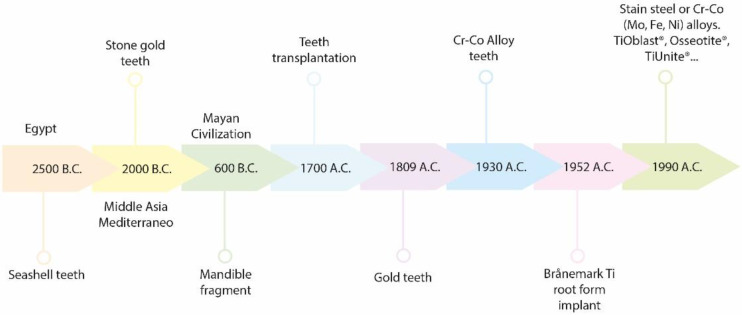
Graphical historical overview.

A historical overview starting in Egypt is presented in Scheme 1. The need to replace natural teeth can be traced back to 2500 BC Egypt where seashells were anchored into human jawbone and stabilized with the use of gold wire. Famous archeological remains have shown that the civilizations in South and North America and regions of the Middle Asia and Mediterranean created artificial teeth using carved stone, shells, bones and gold more than 2000 years ago [4]. Moreover, ruins in Honduras had a fragment of a mandible with three shells carved into tooth shapes, confirming that the Mayan civilization had the earliest known examples of artificial substitutes, dating from about 600 AD [4]. Staple, subperiosteal, and blade vent implants represent the most successful designs of prosthodontic reconstruction that can be found in the early literature, made of noble or base metals. These were but affected by mechanical and biological failures [4,32]. 

In the 1700s, Dr. Hunter mentioned the possibility of relocating teeth from one human to another. During this time in Europe, teeth were extracted from the disadvantaged or from cadavers for allotransplantation [33]. In 1809, J. Maggiolo placed a gold implant into a tooth socket after extraction, but unfortunately an inflammation lead to failure [32,33]. Silver capsules, corrugated porcelain, and iridium tubes were some of the materials employed for dental implants [34].

In the 1930s, Dr. Alvin Strock became known for the successful treatment of shipboard periodontal issues with antibiotics, by provided anchorage and support for restoring teeth. He and his brother used Vitallium (a chromium-cobalt alloy) which at the time was considered a biocompatible material. Implant discovery continually increased during the 1900s and particularly during World War II [32,33]. However, the most important discovery occurred in 1952, when Dr. Per-Ingvar Brånemark, an orthopedic surgeon studying the bone-remodeling process in rabbit femurs, noticed that osseous matter could regenerate and attach to titanium [35]. He defined this phenomenon as osseointegration and used this concept for implant dentistry. In 1978, Dr. Brånemark presented a two-stage threaded titanium root-form implant that was fixed in his patients in 1965 and lasted for 40 years [36,37]. Brånemark Implants^®^ have had a great impact on today’s dentistry [35,38,39]. Many brand devices have been developed (e.g., TiOblast^®^, Osseotite^®^, Steri-Oss Etched^®^, TiUnite^®^, ITI-TPS^®^, Laser-Lok^®^, SLActive^®^) [2], in order to improve the effectiveness of the dental implants and their rate of success. Besides, recent developments including a variety of surface modifications for dental implantology have been made to cope with the fact of being biologically inert, such as acid etching/grit blasting, hydrogen peroxide or acidic treatment, alternative nitride, hydroxyapatite or metal-based coatings among others [40,41,42,43,44,45]. 

## 3. Engineering Strategies in Dental Implantology

The success of dental implants depends on factors such as mechanical overloading, implant-abutment connection design, implant geometry, implant position, bone density, surface finish material of the implant, and micro gap [46]. All of these factors have demonstrable effects on the implant’s integration to the bone tissue and the stress distribution at the bone-implant interface (stress-shielding phenomenon). Osseointegration between the bone and the implant is considered to be the critical factor that interferes in the implant survival rate. Low osseointegration or peri-implant bone loss may cause micro-mobility to the implant and lead to its consequent loss. A peri-implant bone loss of greater than 1 mm in the first year after implantation and greater than 0.2 mm in the following year is considered a failure of the dental implant [46].

Engineering techniques for manufacturing dental implants have played a key role in device design, surface topographies, uncomplicated insertion into the host osseous matter, biocompatibility and costs. Gaviria et al. considered that biocompatibility measures the degree of osseointegration, which correlates to the success rate over a period of time [4]. It is basically influenced by biomaterials composition, implant geometry and surface features [47,48,49].

### 3.1. Biomaterials Composition

The most important properties of an implant biomaterial are: the modulus of elasticity (e.g., 18 GPa for cortical bone); tensile, compressive and shear strength; yield and fatigue strength; ductility (e.g., 8% is needed for manufacturing requirements); hardness and toughness; surface tension and surface energy, and finally, surface roughness [50].

Metals, ceramics and polymers have represented the materials of choice for dental products. Polymeric materials have only been used for fabricating shock-absorbing components because of their low strength. Among ceramics, hydroxyapatite (HA) is the most used because of the great biocompatibility and capability to help the osteoblast activity due to its similar composition to the mineral structure of the bone. However, its low mechanical strength makes it only suitable as a coating material (i.e., plasma-sprayed coatings) [4,51], as discussed later. Something similar happens with bioactive glasses (BGs), a special type of glasses that induce the formation of HA when get in contact with body fluids [52].

Among metals, titanium and its alloys, Ti6Al4V and Ti-β, have been the most commonly used materials because they can osseointegrate. However, all these metals have and materials still have higher modulus compared to the cortical bone (20–25 GPa for cortical bone and 110 GPa for commercially pure titanium and Ti-β). Commercially pure (c.p.) Ti was used in the first Ti implant ever applied, the Brånemark Implants^®^, but they often fail due to high stiffness. Current dental implants have been manufactured to reduce their Young’s modulus. Trueba et al. [53] evaluated the mechanical behavior of superficially modified porous c.p. titanium dental implants fabricated by conventional powder-metallurgy and space-holder techniques. A novel, feasible and repetitive protocol of micro-milling of the implant thread (before powder metallurgy sintering), as well as surface modification treatments (after sintering), have also been implemented. These techniques add porosity and surface roughness to the stiffness and yield strength of implants. Macro-pores concentrate stress locally, and may act as a barrier to the propagation of micro-cracks. Higher rugosity was observed for virgin implants obtained with spacer particles. Concerning the superficial modification of implants, while BG 1393 was the most effective coating due to its greater infiltration and adhesion capacity, chemical etching could improve osteoblast adhesion because it modifies the roughness of the implant surface. Therefore, a new reliable protocol was developed and evaluated to fabricate titanium implants with improved biomechanical and biofunctional response of the interface with the host. 

Lascano et al. [54] investigated the use of a Ti-β alloys, composed of Ti-Nb-Ta-xMn (x: 2, 4, and 6 wt%) and with a lower elastic modulus compared to the c.p. titanium. They included controlled porosity in the implants to reduce more their Young’s modulus, since it was still high (ranging from 50 to 60 GPa). In addition, they include a graphene layer onto the substrates’ surface to enhance their biocompatibility and cell adhesion. The alloy containing 4 wt% Mn favors the presence of the Ti-β phase, and Young’s modulus (8–9.3 MPa) closer to the trabecular bone. Furthermore, Ti6Al4V was developed by the aerospace industry but was applied to biomedical systems for its strength, corrosion resistance, and biocompatibility [55]. These implants have been fabricated in a variety of shapes, such as cylindrical, cone, hollow and screw shapes, and various diameters. Additionally, Ti6Al4V has also been used in orthodontic implants [56], which are temporary anchors. However, some authors reported its long-term toxicity with human osteoblastic and fibroblastic cells [57]. New research approaches tend to avoid the use of elements in the titanium alloys with the potential to cause tissue damage, such as vanadium [58].

Alternatively, zirconium and gold have also been used for the same purpose but, unfortunately, they have demonstrated poor bone-to-implant adhesion [4]. Other materials that have been used for the applications of titanium-based materials in dental implants are based on stainless steel, Co-Cr, Co-Cr-Mo-Ni-Fe or other metal combinations alloys where issues such as cytotoxicity, mechanical, chemical, electrochemical, or biological properties were addressed [59,60,61,62].

### 3.2. Implant Geometry and Surface Features

Implant geometry modulates the bone–prosthesis interaction and distribution forces, changing the surface area and the long-term stability. Thus, cylindrical screw-threaded implants are the most commonly placed [63].

**Figure 4:**
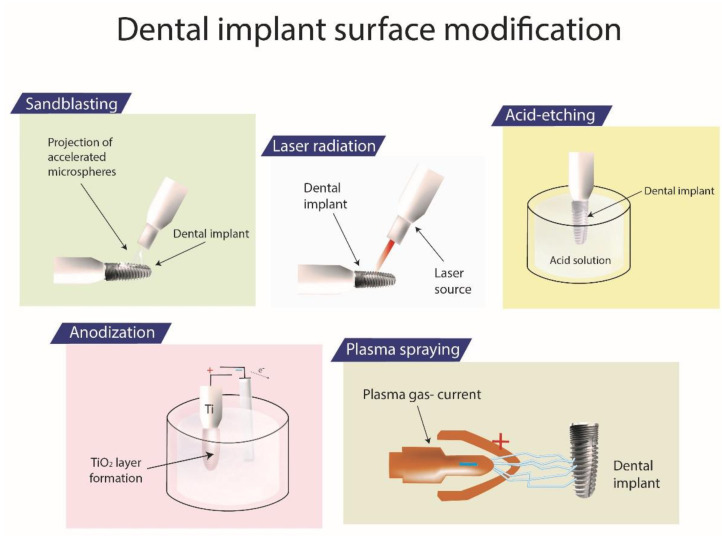
Mainstream engineering strategies in dental implantology.

Surface topography is a very important aspect to consider when designing and manufacturing a dental implant since it directly influences its bioactivity, that is, some of the parameters related to the implant’s survival rate such as the osseointegration or the appearance of bacteria-related infections. The mainstream engineering strategies used to obtain bioactive topographies with surface modifications of the implants are sandblasting, acid-etching, anodization, plasma spraying, and laser radiation (Figure 4).

These techniques have changed the free surface energy, chemical composition, and roughness, which may enhance osseointegration. Rough surfaces (e.g., S_a_ value around 1-2 µm) within hydrophilic composition act as guide locomotion and cellular basement for the adhesion and trigger proliferation of osteoblasts, but high surface roughness can promote the development of peri-implantitis [46,64,65,66] due to bacterial proliferation.

A special mention is made to nanoengineering. The development of nanotechnology, nanoscale modifications and the application of nanomaterials have notably reduced the well-known key issues related to dental implants, such as low osseointegration, the stress-shielding phenomenon and implant-related infections. Nanotechnology has facilitated the implementation of surface modifications, the use of coatings and even the controlled release of antibiotics or proteins. The combination of these approaches has entailed the enhancement of the osseointegration and soft tissue integration of dental implants, as well as their antibacterial and immunomodulatory functions [64].

#### 3.2.1. Sandblasting

Sandblasting implant surface requires particles of various diameters and are one of the most currently commercialized methods for surface modification with the advantages of both topography and wettability [66,67]. The technique of pressured air steam applied to the titanium implant surface generates a macro-roughness due to the projection of accelerated microspheres of TiO_2_, Al_2_O_3_, SiO_2_, or HA. Besides, these four chemical groups act with the same strength as boosting agents for osseointegration [68]. An interesting 4-year clinical observation study showed that titanium implants modified by the sandblasting approach had a higher overall clinical outcome [69]. However, a 20-year follow-up compared a non-modified turned surface device and TiOblast^®^ and suggested these topographies did not improve the bone healing but increased peri-implantitis and implant failure [39]. 

During the sandblasting process, when abrasives are projected to the surface, in order to obtain the optimum macro-roughness, some factors must be monitored [70]. In particular, the choice of the employed particles (i.e., type, size and shape) is a key point, and abrasives must be harder than implant materials to produce roughness [70,71]. Moreover, the distance from the projection gun to the surface, the projection pressure, the saturation time and projection diameter represent important parameters that influence the roughness [70]. Sandblasting has been considered a key treatment to modify implant surfaces with osseo-inductive activity, and to increase the contact angles for the improvement of hydrophilic behavior for osteoblasts adsorption [70,72]. On the other hand, sandblasting induced by abrasives can lead to microbial contamination with the consequence of implant failure [72]. 

#### 3.2.2. Acid-Etching

Osseotite^®^ (Zimmer Biomet, Warsaw, IN, USA) and Steri-Oss Etched^®^ (Nobel Biocare, Zürich-Flughafen, Switzerland) are commercial implants manufactured using the acid-etching strategy [73,74,75]. This technique produces micro-pits surfaces via the immersion of the metallic core in corrosive acids such as HCl, H_2_SO_4_, HNO_3_, and HF [76]. Commonly, the acid-etching erosion takes place after sandblasting and the whole process is considered as the reference surface treatment, referred to as sandblasting and large grit acid etching (SLA) [77]. The Osseotite^®^ demonstrated a success rates of >96% in long-term studies [73,74].

Other authors attempted an acid treatment with a piranha solution—a mixture 3:7 of H_2_O_2_ (30 vol. %) and H_2_SO_4_ (70 vol. %)—not only to confer roughness and therefore, the enhancement of osseointegration to the implant surface, but also to improve its antimicrobial capacity by the chemical linking of silver nanoparticles [78].

Surface features can be tailored by modifying the type and the concentration of the acids used for the acid-etching procedure. Besides, the time of exposition and the working temperature can affect the erosion with the formation of cavities of different dimensions [79]. Titanium surfaces exposed to acid-etching process have demonstrated positive results related to an increase in the roughness and osteogenic response due to the proliferation, adherence, and differentiation of osteogenic cells [80,81,82]. However, in cases of an uncontrolled process, with over-etching or under-etching of the surface, an important variation in mechanical properties, corrosion resistance, or biocompatibility have been observed [83].

#### 3.2.3. Anodization

Anodization is an electrochemical technique used for the oxidation of titanium surface that improves biocompatibility, blood-clot formation, cell adhesion and osteoblast proliferation. During the process, the implant is immersed in acids such as H_2_SO_4_, H_3_PO_4_, HNO_3_ while a current is applied, creating micropores that increase the oxide layer of TiO_2_ in form of anatase. Anatase and rutile have been considered the most important TiO_2_ phases. In particular, in the anatase phase, the unit cell is formed by four TiO_2_ units, where O atoms are coordinated to three titanium atoms and positioned in the same plane in order to form unrelaxed structure [84]. TiUnite^®^ (Nobel Biocare, Gothenburg, Sweden) have been reported to present optimum results, and only 8.2% of the implants in the study were affected by peri-implantitis [85], due to the improvement of the osseointegration.

Anodic electrochemical oxidation is a technique capable of modifying oxide properties, depending on the working electrochemical parameters (e.g., applied voltage, electrolyte composition and concentration, anodization time, bath temperature, stirring) [86]. In fact, the ultrastructural level topographies are a consequence of the migration of the ions throughout the oxide layer, and the thickness of the anodic oxide is determined by Faraday’s Law. The anodization of dental implants was demonstrated to favor blood-clot retention [87], nano-roughness, and osseointegration [88,89]. However, despite the advantageous biological outcomes, the mechanical stability of implants exposed to anodization has represented a challenge to be addressed by many researchers [90], even if good clinical outcomes have been reported [87].

#### 3.2.4. Plasma-Spraying

Plasma-spraying treatment can be associated with SLA to improve biocompatibility and protein absorption because of the additional layer of OH groups. In fact, the implant surface can be modified by the projection of titanium particles injected into a plasma torch at high temperatures, and a film about 30 µm thick is formed by Ti-OH residues [76,91]. ITI-TPS^®^ (Straumann Institute, Waldenburg, Germany) belong to this type of manufacturing in the market [92].

A plasma-spraying process embraces a thick layer of deposition. Hydroxyapatite and titanium are the most commonly used; they are thermally melted and afterwards sprayed on the implant surf area [93]. The combination of hydroxyapatite coating on titanium alloys has been considered very interesting because it demonstrated great biocompatibility and good mechanical properties [94]. Moreover, various strategies have been evaluated in order to obtain the adhesion of hydroxyapatite to titanium implants [95], but only plasma spraying has been actually used in the market [91]. However, this type of coating has demonstrated some drawbacks related to a reverse effect [93], since the bonding strength of hydroxyapatite on titanium alloys decreased by passing the time when the implant is immersed in the simulated body fluids [96]. Recently [97], a suspension plasma spray (SPS) was successful applied for the deposition of hydroxyapatite /gray titania coatings on a titanium surface. Authors reported an improvement in antibacterial properties due to an increase in the number of Magneli phases. Besides, an improvement in the bacterial adhesion was demonstrated because of the hydrophilic properties which correlate to the obtained submicron-sized particles.

#### 3.2.5. Laser Ablation

The above-listed methods can facilitate the formation of macro and microroughness surfaces, while laser radiation creates a nano-topography modification. Laser ablation utilizes the laser sources to produce on-site melting of the metal, as a result of the heating induced by the absorption of the radiation [98]. Thus, a micromatching is formed by the presence of microchannels, prompting a faster healing of the bone [99,100]. This technique can adapt the light frequency, and take advantage of the high energy density and of the all frequencies available. Besides, it is possible to pulse the source and control the reaction time for obtaining the microstructures with increased hardness and corrosion resistance [48,101].

An example of an implant based on this treatment is Laser-Lok^®^ (BioHorizons, Birmingham, AL, USA) [102]. SLActive^®^ (Straumann Institute, Basel, Switzerland) also presents a nanorough surface carried over by blasting and acid etching with a final manufacturing step in which it is flushed with a stream of nitrogen, safeguarding the implant from air and then immersed into a NaCl solution for storage. The benefits of this type of technology include an increase in hydrophilicity in the performance of the biological response and cell recruiting, and faster healing of the bone, with a long-term success rate of around 91.7% [100,102]. 

Other authors have used femtosecond laser [103,104] or directed irradiation synthesis (DIS) [105,106] techniques to obtain micro- and nano-textured surfaces of titanium implants to improve osseointegration by the replication of bone structures, increasing protein attachment and therefore cell adhesion.

Laser ablation has also been used to generate antimicrobial surfaces. Thus, for example, Boutinguiza et al. [107] used this technique to deposit silver nanoparticles on top of c.p. titanium implants.

## 4. Coatings

**Figure 5:**
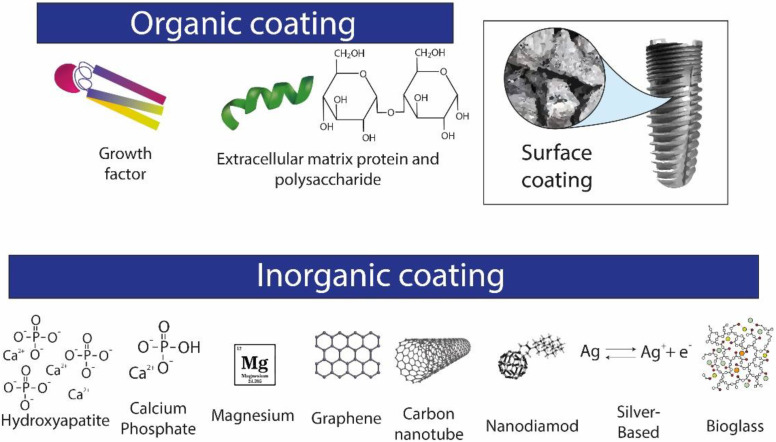
Novel coatings in dental implantology.

The biocompatibility, osseointegration and therapeutic/antibacterial effect of the implant coating have been considered to be the most important factors for the long-term durability of dental implants. Various bioactive materials and local drug delivery techniques have been explored. These novel coatings have been divided into two groups, depending on the nature of the material applied (Figure 5), i.e., organic and inorganic coatings.

Bioactive coatings onto core biometals synergize the bone-healing ability of a bioactive material and the leads to biomechanical advantages of a matrix such as porous titanium. The porous structure focuses stress restrictedly, and it circumscribes small-cracks. HA, magnesium, graphene, grow-factors/specific bone morphogenetic proteins (BMP), and BGs are materials used in functional-coatings technology. The aim in this case is to improve cell–proteins adhesion, mineralization of the implant–host interface, and antimicrobial behavior of the surface layer [53,108].

### 4.1. Inorganic Coatings

Nanostructured calcium, calcium phosphate, and HA) have been the most used implant coatings. They can be applied to a metal implant by hydrothermal deposition or the plasma spraying. These materials release calcium and phosphate ions to encourage the mineralization of the interface tissues and bone-healing [2]. Inorganic coatings also affect how stress is transmitted to the bone during masticatory function and the correct distributions of the forces during the repeated cycles [2].

#### 4.1.1. Hydroxyapatite-Based Biomimetic Coatings

Biomimetic surfaces promote osseointegration in the healing process since they possess a composition similar to the host. HA is the stable biological mineral form of calcium apatite. It camouflages the mineral bone phase and triggers osteoblasts activity for bone formation. HA presents some unique properties because of both its non-inflammatory and non-immunogenic nature. The bioactivity can be boosted by using a micro-arc oxidation technique that allows the formation of a porous HA-coated surface of titanium alloy with positive consequences on biomechanical properties and bone formation [108,109].

Osstem GS-HA III^®^ and Osstem TS III-HA^®^ (Osstem Implant Co., Busan, Korea), which are HA-based devices, have been analyzed and they demonstrated a good balance in the transmission of these forces, but a second work, carried out by the finite element method (FEM), found them to have a weak performance over a long-term period. In spite of that, HA-based coatings showed an optimum rate of success (100%) with the presence of peri-implantitis in 2.2% of Osstem and TS III-HA^®^ implants and, in 1.4% of TSV-HA^®^ [2]. However, it was reported that the plasma-spraying strategy employed for obtaining some HA coatings on metallic implants have presented poor long-term adherence to the core, difficult reproducibility and a variability of the layer thickness and composition and, in addition, can lead to bacterial infections [32]. Other studies highlighted the bioactivity of biomimetic coatings for the reduction of the time required for osseointegration because of both the high wettability of the coated surface and the improved protein adsorption property [109].

Nanotechnology has been applied to HA particles in order to obtain a single coating and combine its bioproperties to collagen, BGs and titanium dioxide for making a biomimetic osteo-matrix. The nanoscale promotes both the specific surface area and adsorption feature, resulting in a better interaction with the host bone [110]. 

A HA coating deposited on metallic pins, which are metal devices used as support of dental amalgam for restoring the tooth [111], has also been studied as bioactive carrier surfaces for the release of an aminoglycoside antibiotic for antibacterial purposes. The tobramycin loaded on the biomimetic coating showed bactericidal activity against *Staphylococcus aureus* in an agar medium that longed 6 days [112]. Other authors incorporated the HA in a polymeric blend to obtain a composite coating with this antibacterial effect. Thus, García-Cabezón et al. [113] deposited HA-containing chitosan nanofibers onto porous titanium implants. The use of this composite to cover the implant’s surface would not only increase the osseointegration but would also prevent the corrosion of the implant. In addition, the demonstrated antibacterial behavior of chitosan and the inclusion of silver nanoparticles would avoid bacteria-related infections. 

#### 4.1.2. Calcium Phosphate-Based Biomimetic Coatings

After the implantation of calcium phosphate-based implants, Ca and P are released into the host tissues promoting the in locus formation of the apatite layer [114]. Enhanced clinical outcomes of implants based on calcium phosphate coatings, with a long-term success rate due to both the promotion of new tissue formation and a wider bone-to-implant contact surface, have been reported over the last years [115,116].

The presence of an inorganic nanotopography in the implant surface affects the cell behavior during the healing process by increasing the gene expression of osteogenic factors, mineralization, adhesion and proliferation. In fact, the coatings of Ossean^®^ (Intra-Lock, Boca Ratón, FL, USA) implants, fabricated by the impregnation of the calcium phosphate nanoparticles within surface microroughness due to a grit-blasting/acid-etching strategy, demonstrated an up-regulation of osteogenesis-related genes since the first week after the implantation and of alkaline phosphatase (ALP) kinase expression (a adhesion/proliferation-related gene) and higher minerals deposition than the dual acid-etched implants [117,118].

Another way to employ calcium phosphate nanoparticles in implant coatings has been represented by combining the acid-etching technique with discrete crystalline deposition (DCD) that allows for the impregnation of the inorganic part during the sol-gel process [119]. Clinical studies have demonstrated that these types of coatings promote osteoconduction with the implant and quite acceptable long-term durability with low bone resorption [120,121,122]. The bone-to-implant contact value demonstrated by Nanotite^®^ (Zimmer Biomet, Palm Beach Gardens, FL, USA), a calcium phosphate nanoparticle-based implant produced using the DCD method, demonstrated important differences in mechanical properties with micro-topographies systems. Micro-roughness implants presented a significantly higher removal torque value than DCD devices but better anchorage to the host bone [122,123].

#### 4.1.3. Magnesium-Based Biomimetic Coatings

The biomimetic property and biodegradable nature of Mg alloys have been explored by researchers over the last few years for the fabrication of new biomaterials that resemble the human bone. Actually, phosphate salts of Mg have demonstrated better resorption kinetics and dissolution rates than calcium salts in clinical trials [124]. In vitro and in vivo studies evaluated this bioactivity which resulted in a better promotion of osteoblasts function and faster bone formation, in comparison to calcium phosphate surfaces, respectively [125,126].

Nevertheless, its alloys have shown some drawbacks with regard their short-term corrosion on the implant interface. Microwave-assisted preparation of magnesium phosphate crystals for obtaining coatings had good outcomes even if the mechanism behind this interaction technique is still under evaluation [127]. Besides, MgCl_2_ and epigallocatechin gallate were employed as a metal–polyphenol network and for osteoinductive coating onto a titanium implant [128]. Moreover, a SLM (selective laser melting) process has been applied to produce porous titanium implant 3D surface with calcium phosphate and doped with magnesium to encourage bone formation. Results demonstrated a higher bone volume increase with magnesium-doped HA coatings than for the control group [129].

In addition, other researchers reported that nanoparticles of magnesium have been studied in dental implantology due to the high level of antibacterial activity against the biofilm formation in *Staphylococcus* implant infections [130].

#### 4.1.4. Graphene-Based Biomimetic Coatings

Graphene application to coatings has been introduced as a new player in dental implants, also because it was isolated in 2004 from the exfoliation of graphite [131]. Graphene is a two-dimensional, one-atom-thick sheets of sp2-bonded carbon atoms, shaped in a hexagonal network. This unique make-up results in some important features such as great electronic, optical and mechanical properties. In fact, the graphene structure presents stability and resistance to degradation caused by mechanical or chemical stresses, differing from Ti6Al4V implants [132]. 

Lately, implant coatings have been developed based on graphene oxide (GO), or reduced graphene oxide, the two-dimensional nature of GO nanomaterial makes it possible to functionalize the large number of oxygen-containing groups on the active surface, with carboxyl and hydroxyl residues [133]. The ultrasonic atomization spraying process has been applied to coat an SLA titanium surface with GO, which showed a good stimulation of cell proliferation and adhesion and effect of the vascular endothelial growth factor on osteoblasts [134]. The coating formed by graphene/HA and deposited on the titanium implant presented a biomimetic behavior due to a better biocompatibility/activity and higher adhesion in comparison to coatings made only by calcium phosphate layer or titanium [135]. 

Moreover, the broad-spectrum antibacterial activity of graphene has been demonstrated by different studies on multi-resistant bacteria and fungi species, and the mechanism behind explored. The effect was due to the damaging of cell membranes through oxidative stress against the microorganisms [136]. In another study, thin layers of GO, incorporated with silver nanoparticles and coated onto the metallic core, showed activity against *Streptococcus mutans* and *Porphyromonas gingivalis* [137]. In addition, it has been demonstrated that the number of GO layers increases antibacterial and osteogenic activities, as shown by the minocycline-loaded GO implant that killed *Staphylococcus aureus*, *Streptococcus mutans* and *Escherichia coli* with no side effects on the human gingival fibroblasts [138,139]. In dental implants, GO hybrid materials have been tested using different concentrations of GO soaked in chitosan. These hybrid materials were deposited onto Ti substrates, demonstrating osteoblast proliferation and biofilm inhibition [140]. In Y-ZrO_2_ implants, GO coatings improved the mechanical properties of samples, such as the bending strength and the fracture toughness, while increasing cell adhesion, proliferation and growth [141]. 

#### 4.1.5. Carbon Nanotube-Based Coatings

Carbon nanotubes have gained momentum as biomaterial for medical applications since they present both chemical durability and biomechanical and electrical properties. They can be used as coatings for titanium cores covered with collagen, promoting surface roughness, cell proliferation and adhesion [142]. Moreover, the bone healing activity of these coatings has been well demonstrated by culturing multiwalled carbon nanotubes on rat primary osteoblasts. Alkaline phosphatase activity and calcium and osteopontin contents were higher than the non-coated group of the control. Eventually, after 28 days of the implantation of multiwalled carbon nanotubes with osteoblasts, the presence of the bone matrix was shown in the pores of the honeycomb structure [143]. Recently, an in vitro evaluation of multiwalled carbon nanotube reinforced nanofibers for dental application was performed. Multiwalled carbon nanotubes were incorporated into electrospun nanofibers of nylon-6 that were later added to a polymeric resin in different concentrations. The most promising composite was the one containing 2.5 or 5% of nanofibers due to their adequate flexural strength associated with reduced film-thickness [144]. In order to solve some drawbacks related to HA coatings, researchers have proposed HA-carbon nanotube composite coatings on a titanium core, obtained by aerosol deposition. These systems showed no microcracks, and led to an improvement in mechanical properties such as hardness and the elastic modulus. Besides, they presented a better clinical outcome by promoting cell proliferation and the alkaline phosphatase activity than coatings made only of HA and titanium [145]. Kim et al. demonstrated that the addition of carbon nanotubes to polymethyl methacrylate resins prevents bacterial adhesion without cytotoxicity to oral keratinocytes [146].

The nanoscale size and the large surface areas of carbon nanotubes and graphene have favored their use for making biomaterials for clinical purposes and regenerative medicine. However, since they interact with proteins, nucleic acids and host cells, their cyto-compatibility has been explored during the last few years and it is still controversial [147].

#### 4.1.6. Nanodiamond Coatings

Nanodiamonds are formed by a unique structure and surface, containing oxygen-carrying groups and charges that have been considered responsible for the antibacterial activity of the coatings (e.g., *Escherichia coli*). The biocompatibility of nanodiamonds-based coatings has been evaluated since their interaction with biological living organisms. A study of this type was presented by Vaitkuviene et al. [148] who observed the effects on neural cells, concluding that the thin layer of oxygenated and boron-doped nanocrystalline diamond increased cellular proliferation and adhesion. In general, particles a size of 100 nm or more presented better results than smaller ones. In addition, the current literature determining the mechanical properties and bond strength of nano-diamond coatings on dental implants is scarce and requires further research. In addition, the biocompatibility of nanodiamonds has been explored by in vivo studies, but controversial results have been obtained, and further investigations are required. In dental implantology, the literature is still incomplete concerning mechanical properties or the bond strength of the coating to the implant [109].

#### 4.1.7. Silver-Based Coatings

**Figure 6:**
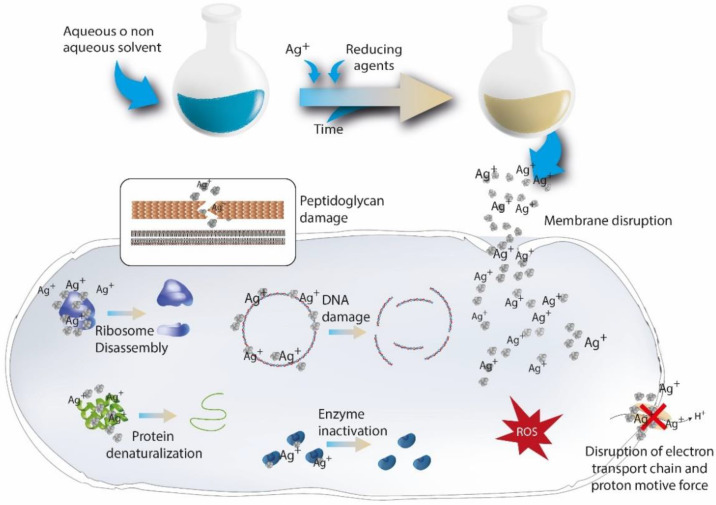
Synthesis and proposed mechanisms of action of Ag^+^ and silver nanoparticles in bacteria.

Since the inorganic nature of silver as element, its activity as antibacterial agent was introduced in this section. Inorganic-based coatings with an effect against microorganisms have demonstrated better properties such as chemical stability, thermal resistance and long-lasting activity, than common organic agents. Besides, silver has exhibited oligodynamic antimicrobial activity that consists of bactericidal-bacteriostatic effect at very low concentrations. This agent has proved a broad spectrum of action against Gram-positive and Gram-negative bacteria within a low propensity to develop bacterial resistance, and a successful inhibition against the polymicrobial colonization [149]. The biocidal effect of silver is mainly due to Ag^+^ ions which alter the permeability of cell walls, interfering with vital proteins and triggering DNA condensation (Figure 6). This behavior has allowed silver-based coatings to be effective against the typical dental implants-associated pathogens (e.g., *Streptococcus mutans*, *Staphilococcus aureus*, *Streptococcus Oralis*, and *Actinobacillus actinomycetemcomitans*). 

Antimicrobial and osteogenic properties of Ag^+^-implanted stainless steel have been explored with in vitro and in vivo tests. The silver-sourced plasma immersion ion implantation (Ag-PIII) was used for modifying the surface of stainless steel, which was doped with silver nanoparticles. The Ag-PIII treatment promoted anti-infective activity and demonstrated the osteogenic differentiation of bone marrow stromal cells [150]. The antimicrobial effect has also been explored for coatings made of HA–silver, plasma sprayed silver-implanted HA and silver loaded gelatin microspheres, incorporated into porous titanium that controls the metal release in situ [149].

The need for performing silver coatings by functionalizing porous titanium surfaces is based on their high rate of fail after implantation, mainly due to the presence of infections. Silver nanoparticles have been proposed as an important alternative to the common silver coatings since they induce a convenient prolonged release of Ag^+^ ions. Moreover, silver nanoparticles are adsorbed onto the bacteria membrane, which is dependent on Coulomb gravity, triggering protein coagulation [151,152] (Figure 6). A combination of a porous titanium implant, for the reduction of the stress-shielding phenomenon and enhancement of osseointegration, and a silver nanoparticle-coated substrate, for bacterial inhibition, have been tested. The deposition of nanoparticles was achieved by chemical bounding after a silanization reaction to the hydroxylated surface. In order to evaluate the outstanding properties of the implant, microstructural features and anti-infective effects against *Staphylococcus aureus* were considered [78,153]. Other authors highlighted the importance of a relevant nanocomposite coating for the antimicrobial properties by exploring the activity of surfaces formed of polysaccharide 1-deoxylactit-1-yl chitosan and silver nanoparticles on methacrylate thermosets. Moreover, the adequate level of ions content, delivered at first, was able to kill pathogens quickly and prevent the development of drug resistance.

However, over the topic remains controversial. On the one hand, the inactivation of the silver antimicrobial effect in human fluids and, on the other hand, the low cut-off level of Ag^+^ ions required for cytotoxic activity, are very important aspects in the case of nanoparticles since the release is prolonged. Even so, the capability of silver as a biocide in absence of cytotoxic effect on osteoblasts and epithelial cells has also been demonstrated [154]. Silver electrodeposited on tricalcium phosphate (TCP)-coated titanium implants have been reported for the expected bactericidal effect without cytotoxicity since the optimal silver release. In the same way, the cold-spray coating technique utilizing polyether ether ketone (PEEK) and the chitosan–copper composite for creating antimicrobial implant coatings have presented similar results [109].

#### 4.1.8. Bioactive Glasses

**Figure 7:**
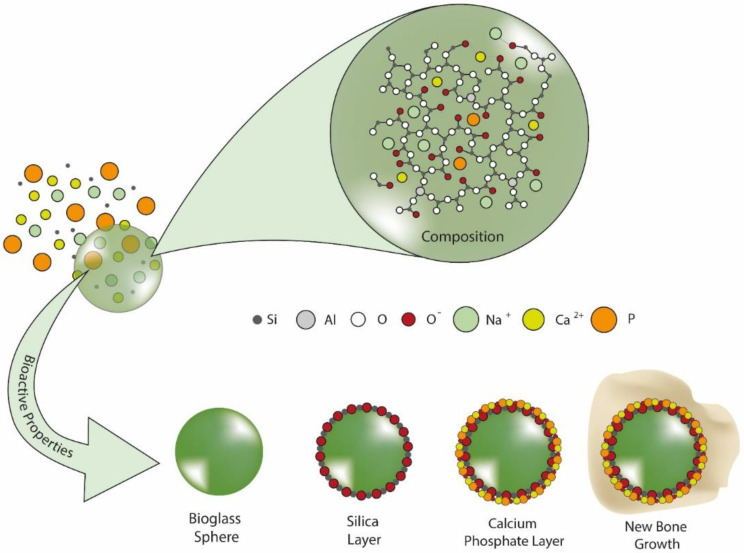
Schematic of the different steps that lead to the generation of HA on top of the bioglass particle. Initially, there is an ion exchange on the BG surface that leads to the leaching of calcium and sodium cations from BG, which is replaced by H^+^ from the medium. The acidification of the BG surface entails the liberation of silicic acid, producing a silica-gel interface that absorbs Ca^2+^ and phosphate anions. Finally, the amorphous calcium phosphate layer generated crystalizes to HA.

BG for metallic substrates coating has been considered, in comparison to other surface modifications, more effective and efficient in terms of a high Young’s modulus (preventing the stress-shielding phenomenon) and osseointegration (stimulating bone regeneration). In fact, BGs have indicated unique properties that provide a versatile tool, based on the possibility to change their composition, for clinical application, such as bone regeneration and drug delivery. Professor L. L. Hench gave the definition for BGs as “special glasses able to bond to bone or even to soft tissues, without any rejection” [52,155]. In general, they have demonstrated the capability to form precipitates of HA on the glass substrate (Figure 7), when exposed to contact with human fluids, resulting in bonding to the host bone. The biomimetic ability to produce HA resembling the natural one has been demonstrated to promote the osseointegration of the implant. Besides, they also showed osteoinduction properties within the bone formation at the implant–device interface [156]. BG porosity and roughness, modulated by deposition techniques, are important features since they affect bioactivity. Moreover, the chemical composition, which is highly variable, is a key factor determining their final properties. According to it, they can be classified into different types, such as silicate-, borated- and phosphate-based BG coatings [157]. Silicate-based BGs (e.g., BG 45S5 and 1393) have been the most used. In fact, Torres et al. demonstrated the key role of the implant pores in the anchoring of a bioactive coating. They investigated the coating of diverse c.p. titanium implants with an original bilayer of BGs, 45S5 and 1393, arranged according to their specific characteristics to maximize the osseointegration. In this sense, the 1393 BG layer was in contact with the porous titanium substrate because it presents a theoretically better adherence to the titanium, while the 45S5 BG layer was surrounded by the host bone tissue since it promotes higher bioactivity, demonstrated by solid NMR. The role of the pores was a crucial issue in the anchoring of the coating, both in porosity percentage (30 and 60 vol %) and in pore size range (100−200 and 355−500 μm). The results revealed that the substrate with 30 vol % of porosity and a range of 355−500 μm pore size, coated with this novel BG bilayer, presented the best combination in terms of mechanical and biofunctional properties [155,158]. The bioactivity enhancement induced by the presence of BG was also utilized to prepare biphasic implants required when both bone and cartilage tissues are damaged. In this sense, Torres et al. [159] described the deposition of BG 45S5-gelatin coatings on porous titanium implants, whereby the BG-polymer composite coating would replace the cartilage and the porous titanium implant would substitute the bone tissue. The preliminary results exhibited a promising route to fabricate composite-coated porous titanium implants as potential candidates to develop alternative treatments for diseases in which tissues of different nature need to be perfectly joined.

### 4.2. Organic Coatings

#### 4.2.1. Growth Factors-Based Coatings

**Figure 8:**
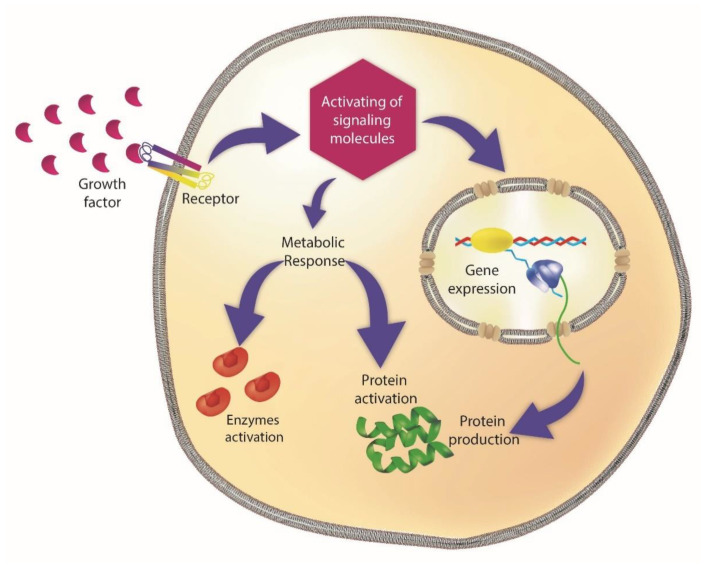
Schematic representation of growth factor activity on protein and enzymes activation, and gene expression in human cells, inducing proliferation: growth factors attach to the receptor, which initiate a signaling cascade. This cascade activates a metabolic that induces enzymes and proteins activation. It also changes gene expression that transcripts and translates to proteins, such as enzymes.

Growth factors are a group of molecules involved in the cell division and tissue proliferation. Each growth factor recognizes a specific membrane receptor and their union initiates or inhibits cell division (Figure 8). Main growth factors used in implant coatings are bone morphogenetic proteins (BMPs) and vascular endothelial growth factor (VEGF).

VEGF is a signal protein that has shown to activate gene and protein expression of vasculogenesis and has been found, during in vitro experiments, to enhance primary rat osteoblast proliferation and to increase alkaline phosphatase (ALP) activity. In addition, the in vivo experiment showed an important improvement in the activation of osteoblasts and endothelial cells [108].

BMPs are proteins with a key role in inducing bone and cartilage formation by the regulation and promotion of osteogenic and bone mesenchymal stem cells. These characteristics have led BMPs to be increasingly applied in dental coatings. In fact, the high demand of these molecules has led to the use of recombinant techniques to obtain them in a good yield. In 2007, the Food and Drug Administration (FDA) approved the recombinant human BMPs (rhBMPs) for therapeutic uses in dentistry, including rhBMP2 and rhBMP7 [108]. Currently, rhBMP2 is commercially available and has been used for bone regeneration in dental implantology. Although its delivery in the protein form has been tested, best results were obtained when rhBMP2 gene delivery was investigated. In this sense, dosage issues were found in the first case and safe and effective dosage have not been determined yet. Meanwhile, rhBMP2 gene delivery displayed a synthesis of the protein for weeks to months during in vivo experiments, depending on the vector [160]. However, the use of viral vectors is an important concern when translating these techniques to the clinical practice, although some trials in dentistry have been ongoing. When applied soaked in a poly(ethyl acrylate) coated titanium surface, it showed excellent osseointegration when included in very low concentrations [161].

A study regarding the effect of anodized implants coated with combined rhBMP2 and recombinant human VEGFs on vertical bone regeneration in the marginal portion of the peri-implant showed alveolar bone regeneration and enhanced bone-implant contact in the microthread [162]. 

#### 4.2.2. Extracellular Matrix Proteins and Polisaccarides

The accumulation of extracellular matrix (ECM) proteins is another approach tested to enhance the biocompatibility on implant surfaces. During the growth phase of bone integration, fibroblast growth factor stimulates fibroblasts to secrete ECM proteins, including elastin, chondroitin sulfate collagen, fibronectin, hyaluronic acid, and other proteoglycans [108]. As examples, Kellesarian et al. [163] demonstrated that the application of collagen-chondroitin sulfate as an implant coating clearly increased osseointegration by promoting bone formation in the implant–bone interface. The use of mussel adhesive protein was tested by Yin et al. [164]. These proteins enhance osseointegration promoting the differentiation of bone-forming cells as well as favoring cell adhesion and proliferation. Regarding the improvement of the mechanical properties caused by a rapid osseointegration, Raphael et al. [165] demonstrated that the use of elastin-like protein for coating implants in a rat tibia and femur led a reduction in the implant micromovements related to a deficient force load. Moreover, the experiments carried out by Sabino et al. [166] showed that the inclusion of hyaluronic acid on polyelectrolyte multilayer coatings improved osteogenic differentiation of adipose-derived stem cells and bone mineral deposition.

### 4.3. Antibacterial Strategies of Coatings

**Figure 9:**
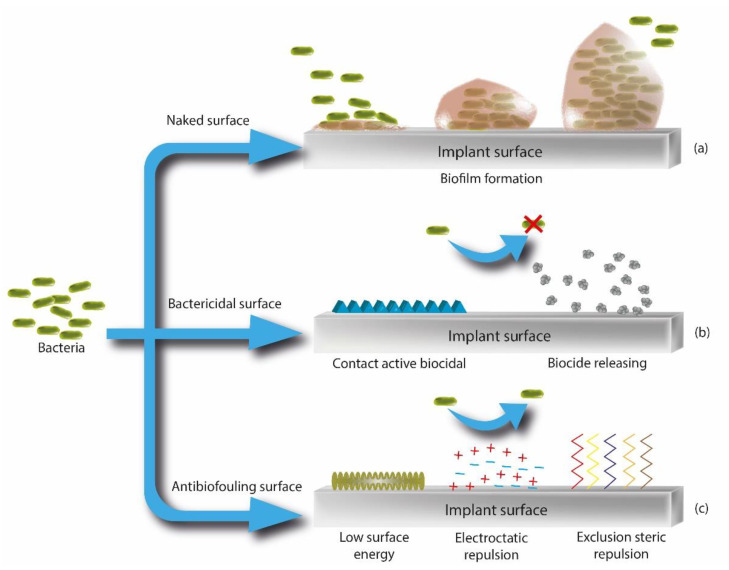
Bacterial interactions with implant’s surfaces: (**a**) on naked surface (**b**) on surface with killing activity (**c**) on surface with repelling activity.

Dental-implant failure has been commonly associated with the presence of infections as postoperative complication of restoration. As mentioned in the introduction of this review, bacteria colonization coexists with biofilm, which protects microorganisms on the implant surface. Gram-positive bacteria, (i.e., *Staphylococcus epidermidis*, *Staphylococcus aureus*, and *Enterococcus* spp.) and Gram-negative bacteria, such as *Pseudomonas aeruginosa*, represent the most common causes related to dental implants infections. In order to develop effective infection-control actions, researchers have explored new coatings that interfered with the two staged mechanism of bacterial adhesion, inhibiting biofilm formation. In fact, when a biofilm layer is formed, the eradication of the infection can be very difficult due to the presence of the exopolysaccharidic matrix, which reduces both the penetration of antibiotics and the vascularization of the site (Figure 9). Thus, the prevention of biofilm formation by selecting antibacterial surfaces, with repelling or killing activity (Figure 9), has been considered a key point to obtain a high rate of implant success during a long period of time after the implantation. Depending on its treatment, implants lead to biofilm formation on their surfaces if these are naked, due to the high biocompatibility of the materials commonly used to fabricate them. However, they could be treated to kill bacteria by a biocidal agent which can be chemically linked to the surface or sustainedly released from it. In addition, antibiofouling treatments could be applied to avoid bacterial adhesion to surfaces, for example, causing steric or electrostatic repulsions or low surface energy by modifying surface topography. Researchers have tried to produce systems for a correct release of drugs and avoiding their excessive application, which induce the growth of drug-resistant bacteria [108].

In this section, different novel dental implants coatings with antibacterial effect are described in detail, such as drug-releasing systems, antimicrobial surfaces with no-drug release and antifouling biomaterial strategies.

#### 4.3.1. Drug-Releasing Coatings

Taking into account all these facts, the development of original coatings able to release drugs from the implant into the host oral cavity has been considered a therapeutic challenge for researchers over recent decades [167,168,169]. During the delivery process, the active molecules can be transferred from the biomaterial to the environment with a controlled approach [170]. In fact, the coatings of the implant or its bulk structure can allow drugs to be eluted to the environment by diffusion, osmotic pressure, and via matrix degradation for a period of time [171]. 

In particular, it has been reported that molecules are released from implants through four main mechanisms, namely diffusion-controlled, solvent-controlled (i.e., osmotic or swelling phenomenon), chemically controlled (i.e., polymer biodegradation and bond cleavage of the drug-biomaterials association) and pH-sensitive mechanisms [170]. In addition, dissolution, diffusion, portioning, erosion and molecular interactions have been explored as other possible mechanisms for drug release [172,173,174]. Enzymatically degradable coatings and soluble biopolymers have been also developed with this aim [175,176]. A proposal of classification has been provided by Stewart et al. [177], and drug-delivery devices were grouped into two main categories, of passive implants and active implants. Passive systems were considered biodegradable or non-biodegradable, and the active implants subgrouping relied on the type of energy/method driving to release the drug. Once the passive implant is installed, the delivery is governed only by the chemical-physical features of the material, fabrication method and drug formulation. Conversely, in the active release, the whole process is controlled by mechanical, electrical, magnetic, laser or other mechanisms. In addition, since commonly metallic devices have been employed in clinical implantology, researchers have suggested that the trigger for the drug release should be based on external stimuli, providing the required dosage for the healing effect [178].

The elution time of the active molecule is also affected by surface topographies of the implants and the drug load. Biomaterials such as polymeric matrices, hydrogels and nanoporous matrices have been evaluated for this purpose [179,180,181,182,183,184,185]. Types of carrier-based drug delivery biomaterials have included dendrimers and micro- and/or nanoparticles (e.g., micro- and nanospheres, nanofibers and nanocapsules) [186]. In addition, due to the increase in technological advancement, ceramic substrates have also been used as systems for controlled drug release. In fact, a nanometer scale led to the development of mesoporous silicas devices for better control over molecule loading and release kinetics [187].

The desired properties of an implant-coating drug delivery system include a biocompatible material without secondary side effects both locally and systemically, minimizing complications related to the mismanagement of a drug delivery dose but with high bioactivity to promote osseotintegration. Various modifications of implant healing coatings for in situ infections have been explored by incorporating antibiotics, antimicrobial peptides, and antiseptics [109]. Additionally, antibiotics have been loaded on the implant surface to destroy pathogens, preventing acute or chronic infections, but avoiding repercussions for eukaryotic cells. Dipping or spray-coating methods have been investigated for the incorporation of antibiotics to the implant with the aim of obtaining a therapeutic drug release coating in order to replace the use of systematic agents, reducing toxic effects [188,189,190,191]. Gentamycin belongs to broad-spectrum antibiotics, and it has been widely applied in dental implantology for the development of therapeutic coatings with HA [192,193]. A biodegradable gentamicin-polylactic acid-coated implant was studied in rats. It was demonstrated that the addition of 10% gentamycin to the polylactic acid surface adds favorable activity against implant-related infections [194]. In addition, another in vitro study with nanotubes, made of titanium throughout anodization of the bulk material and loaded with gentamycin, were capable of promoting osteoblastic activity, and had great effects against bacterial adhesion [195]. Likewise, tetracycline-loaded chitosan coatings have exhibited an improvement of both the bond and retention of blood clots on the host bone, promoting osseointegration [196]. Other authors evaluated gentamicin-based coating outcomes by employing antibiotic-loaded fibers obtained by electrospinning. They demonstrated an optimum activity for eradicating peri-implantitis-associated bacteria and biofilm development [167]. Another work confirmed the promotion of early bone healing with tetracycline-incorporated polymer nanofibers by observing that the expressed ALP kinase was relatively higher than for other systems [197]. However, tobramycin has been loaded instead of gentamicin on implant surfaces, reducing ototoxic effects, and local distribution to the skull at high doses [198]. 

A reduced adherence of *Staphylococcus aureus* to the titanium implant was obtained by the slow release of the agent from a coating formed of vancomycin loaded silica sol–gel film [199]. Moreover, to formulate vancomycin-loaded coatings with long-lasting anti-adherence activity, the drug was covalently attached to different implant surfaces. Results showed that released vancomycin had the same activity as the standard drug against *Staphylococcus aureus*, independently of surface morphology. This last aspect indicated that the topography can be tailored to improve the cellular response or osseointegration, with no effect on the total peptide released and positive therapeutic outcomes [200]. Synergic effects were obtained when researchers loaded polymers such as chitosan and hyaluronic acid with vancomycin-encapsulated titanium nanotubes. These polymers were functionalized with catechol, reducing the concentration of reactive oxygen species, and consequently inflammation, but supporting osseointegration. In addition, it was reported that bacterial hyaluronidase was able to digest hyaluronic acid, producing a slow release of the antimicrobial peptide, which inhibited *Staphylococcus aureus* attachment in vitro, and promoted the bone healing in an in vivo experiment [2]. Besides, Cometa et al. took advantage of hydrogels as versatile candidates for drug controlled release by developing vancomycin and ceftriaxone-modified poly(ethylene glycol diacrylate) hydrogel coatings for titanium dental implants. These coatings displayed remarkable antibacterial activity against methicillin-resistant *Staphylococcus aureus* [198].

Doxycycline has been commonly employed for the formulation of antibiotic-loaded coating since it suppresses bacteria growth and prevents both peri-implant inflammation and bone resorption. In vivo studies showed that HA coatings treated with doxycycline reduced the progression of peri-implantitis [201]. Moreover, dental implants formed of titanium nanotube surface coated with polylactic-*co*-glycolic acid and doxycycline have shown to provide pH-sensitive systems for a controlled release of the drug [202]. *Porphyromonas gingivalis* growth has been demonstrated to be inhibited, during a 28 day period interval, by nanotubes loaded with doxycycline on the dental implant surface [203]. In addition, doxycycline-based coatings were found to prevent microorganism colonization and consequently control of peri-implant mucositis and peri-implantitis [204].

Nanospray drying technology was used for preparing tailor-made biocompatible nanocoatings of polylactic-co-glycolic acid with antibacterial activity due to the presence of norfloxacin. The technology was applied on titanium discs as material for dental implants, tested in vitro, and showed great activity against bacterial accumulation (i.e., 99.83% of reduction in the number of viable colonies) [191]. Bacitracin has been covalently fixed to dopamine coatings, and presented strong activity against macrophage spreading, *Staphylococcus aureus* and its methicillin-resistant counterparts, but promoted adhesion, proliferation, and osteogenic differentiation of the human bone marrow mesenchymal stem cells [205].

Titanium surfaces have also been functionalized with quercetin, demonstrating that flavonoids promote osteogenic activity. In fact, they increase alkaline phosphatase function and promote mineralization of primary human mesenchymal stem cells, but prevent peri-implantitis since they decrease the release of the inflammatory mediator PGE2 [206]. Likewise, TiO_2_ nanotubes have been loaded with propolis, which contain high levels of flavonoids, and tested in rat mandibles for dental-implant applications. Results confirmed that this type of coating improves cell proliferation and differentiation within a proper osseointegration [207].

Moreover, chlorhexidine has been considered a broad-spectrum antimicrobial and antifungal agent, and it has been used for the treatment of odontogenic infections. It showed satisfactory antibacterial results when the drug was loaded into microporous silica coatings by diffusion, which avoided the burst release of the drug [190]. In another experiment, titanium surfaces were coated with chitosan containing 20% tetracycline or 0.02% chlorhexidine digluconate. The results reported that the coatings released 89% of the tetracycline in one week but 100% of chlorhexidine in two days. In addition, released tetracycline inhibited bacterial growth with no cytotoxicity to host cells, and the chlorhexidine presented activity against microorganisms at up to two days, but it was toxic to humans since the first day [196].

It has been reported that halogenated furanones, extracted from the red alga *Delisea pulchra*, have strong effects against various pathogens. The unique activity is based on their structure, such as bacterial *N*-acyl homoserine lactone, which renders halogenated furanones less able to induce drug resistance. In fact, *N*-acyl homoserine lactone acts as signal molecule in bacterial communication to control population growing and biofilm formation. A novel coating formed of nanoparticles containing a halogenated furanone compound was fabricated for the prevention of peri-implantitis issues. The study concluded with good results on the preventive effect at the early stage [208].

A slow release of minocycline from microspheres was recently reported during an in vivo experiment. Chitosan-coated alginate and poly(meth)acrylate-glycerin were explored as local minocycline microsphere carriers for the treatment of peri-implant mucositis. Chitosan-based microspheres showed longer effect as carrier, and better bacteriostatic activity than the poly(meth)acrylate-glycerin ones. Nevertheless, authors claimed that the longer drug sustainability did not lead to improved treatment [209].

#### 4.3.2. “No-Releasing” Coatings

The development of biopolymers with antibacterial effects has represented a feasible option in the field of implants coatings over the last years. In fact, their ability to act as biocides, while avoiding the release of substances, prevents the toxicity arising from the diffusion of the low molecular weight drugs through the polymeric matrix. In addition, these polymers usually present longer-term activity. Thus, biopolymers, due to the intrinsic features, have been commonly used in implants coatings to mitigate, combat and/or eradicate infections, inhibiting bacteria adhesion and biofilm formation [210]. Francolini et al. gave a definition for these family of coatings: “antimicrobial polymers are usually referred to as polymeric biocides, if obtained from polymerization of bioactive repeating units, or biocidal polymers, when the whole macromolecule is bioactive” [198]. However, even if the polymerization of active substances induces a decrease in toxicity, it also reduces the antimicrobial effect. It has been reported that vancomycin-modified poly(ethylene glycol methacrylate) resulted in a polymer that was sixfold less effective than the low molecular weight drug [211]. On the other hand, biocidal polymers present many advantageous properties such as being non-volatile, chemically stable and unable to pass through host skin [212].

Polycationic polymers are commonly employed since they can establish bonds with the membrane of microbial cells. The mechanism can be briefly described as follows: after the absorption, the polymer penetrates into the cell wall and interacts with lipid or protein of the membrane, thereby inducing disassembly which results in leakage of intracellular small molecules, degradation of proteins and nucleic acids, and eventually cell wall lysis [213]. In this regard, chitosan and carboxymethyl chitosan, which are polysaccharide of natural origin, formed of N-acetylglucosamine and D-glucosamine that vary in composition, sequence, and molecular chain length, have been the most extensive antimicrobial polymers explored among natural cationic polymers. They are considered agents with a broad spectrum of activity due to their killing effect against Gram-positive and Gram-negative bacteria [214]. However, they have presented some drawbacks due to unspecific inhibition of osteoblasts and bacteria fixation. Thus, they have been functionalized with agents with adhesion activity such as HA [215], bone morphogenetic protein-2 [216], ALP [217], silica–chitosan hybrid materials [218], and chitosan-58S BG nanocomposite [219]. Moreover, it has been demonstrated that the covalent grafting of chitosan to substrates of titanium through a glutaraldehyde linker were more effective than chitosan used in a solution as a killing agent [220], and in the prevention of biofilm formation by *Pseudomonas aeruginosa* and *Staphylococcus aureus* [221].

Silk is considered a protein polymer and it has been explored as a biomaterial for dental implants. It has been studied for tissue regeneration, alone or in a composite materials (e.g., silk-HA composites). Tissue engineering and regenerative medicine have demonstrated the biocompatibility and low immunogenicity of this natural polymer, so it has been considered a possible strategy for coating applications [64,222]. In addition, antibacterial feature can be included in silk-based coatings, as in the case of silk nanocomposites containing silver nanoparticles [223]. The analysis of the blend showed the presence of nanoTD particles dispersed in the SF blend, which promoted the capacity to control bacterial growth when compared to pure SF membrane [224]. 

Photoactive coatings, based on metal oxide nanoparticles, have represented a second strategy of non-releasing surfaces with antibacterial effect. These systems have demonstrated to generate reactive oxygen species (ROS) in the presence of ultraviolet or visible radiation. ROS damage carbohydrates, lipids, proteins, and DNA, and induce bacterial cell death. Furthermore, TiO_2_, CuO and ZnO nanoparticles showed antibacterial effect due to their ability to promote the presence of oxidative stress linked to photogenerated ROS [225]. In addition, since nanosystems provide much more surface area than bulk materials, they are more exposed to the radiation with the consequence of producing more ROS. Additionally, TiO_2_ represents the most used material employed in this field. In fact, titania was found to promote osteoblast adhesion and proliferation [226]. Moreover, TiO_2_ coatings were able to suppress adhesion of *Streptococcus mutans* and killing effect against *Streptococcus mutans* and *Porphyromonas gingivalis* [227].

#### 4.3.3. Antifouling Coatings

Antifouling coatings have been gaining momentum in dental implantology due to their great activity for repelling microorganisms. Hydrophilic polymers, zwitterionic materials, and superhydrophobic materials have been employed for this purpose.

Poly(ethylene glycol) (PEG) has been the most explored hydrophilic polymer to obtain coatings with antifouling properties. In fact, PEG chains act against protein adsorption through a steric-repulsion mechanisms, and also due to the formation of a barrier made of structured water associated with PEG [228]. This component mechanism was investigated for the first time by Jeon et al. in the early 1990s, when it was demonstrated that the interaction between the surface and proteins was inhibited by the repulsive electric forces arising from the compression of the highly mobile PEG, which required the removal of water from the hydrated polymer. Besides, the presence of this tightly bound water layer interacting with the PEG was considered a physical obstacle for protein and pathogens [229]. Several strategies were used to immobilize PEG on the biomaterials surface, namely self-assembly, physisorption, silanization, electropolymerization, plasma polymerization and pulsed electrodeposition [228,230].

The use of long polymer chains favored more efficient surface coverage, arising from PEG coatings formed by self-assembly. When the process of self-assembly took place on a surface, highly ordered low-dimensional structures can be spontaneously formed onto the surface, the so-called self-assembled monolayers (SAMs) [198]. In fact, the self-assembly process has been commonly considered as the gold standard to obtain tailored/ordered structures in other fields for clinical and toxicological purposes [231], and it was defined by Accioni et al. as “the phenomenon by which isolated components organize autonomously and spontaneously into ordered and/or functional structures” [232]. Moreover, self-assembly provided short PEG chains with protein-resistant activity in the work of Prime and Whitesides, who developed SAMs formed of alkanethiolates displaying short PEG chains [233]. Recently, SAMs for dental implantology have been considered an easy and precise way to tailor surface properties since these systems are formed of well-organized organic structures with the unique feature of modifying chemical properties of the interface at the molecular scale. Besides, in order to develop new biomaterials, these properties can be tailored during synthesis for obtaining feasible study systems for the interaction between surfaces, proteins, and cells [234].

The stability of PEG coatings was evaluated and showed good results under sterilization processes and exposure to physiological conditions in phosphate-buffered saline (PBS) [198]. Controversially, PEG presented some limitations due to the rapid auto-oxidation [235] when exposed to oxygen, metal ions and some enzymes, which can affect the long-term durability of the coating [236].

Zwitterionic materials represent the novel generation of polymers with antifouling properties over the last years. They were prepared with biocompatible biomaterials for the development of coatings, resins, adhesives, cements, composites, varnishes, and sealants [237,238]. Their structure results in an overall neutral system because of the presence of an equal number of positively and negatively charged functional groups. In addition, zwitterionic materials have been considered to simulate the biologic membrane of phospholipids, because they are able to arrange themselves in a bilayer with the hydrophilic groups positioned outwards, and the hydrophobic chains positioned inwards. Moreover, they have also been used as super hydrophilic protein-repellent polymers due to the presence of hydrogen-bond acceptor groups rather than donors, which induce a hydration shell trough electrostatic interaction. In this way, fluids with a high protein content, such as saliva, are repelled and biofilm formation can be prevented [239].

Coating of a surface with the zwitterionic 2-methacryloyloxyethyl phosphorylcholine (MPC) co-polymer has been demonstrated to reduce the retention of the pathogens *Staphylococcus aureus*, *Streptococcus mutans*, *Pseudomonas aeruginosa*, and *Candida albicans*, attributed to the superhydrophilicity of MPC-coated. Other researchers reported that MPC coatings decrease the adhesion on plastic coverslips and HA disks by common microorganisms and peri-implant pathogens. In addition, MPC have been combined with quaternary ammonium compounds (QACs) to obtain a synergic clinical effect. It was demonstrated that the contact-killing activity of QACs can be reduced in presence of salivary pellicle, which is reduced by the protein-repellent effect of MPC [240]. However, in previous studies, MPC-based coatings compromised the mechanical properties of the bulk dental material. Thus, some authors reported sufficient results when MPC was photochemically added onto the surface of filled composite resins during the dental procedure [241]. A new polymer made of MPC, *n*-butyl methacrylate and 2-metahcryloyloxyethyl-4-azidobenzoate was explored as an antifouling coating. It showed great ability to inhibit both the formation of plaque on the coated implant, and to withstand chemical and mechanical stressors until to two weeks. Moreover, it also demonstrated a reduction in *Fusobacterium nucleatum* and *Streptococci* spp. during 5 h in a crossover trial [242].

Superhydrophobic materials have been explored as coating (contact angle greater than 150°) for dental implants because they showed great antibiofilm properties. A superhydrophobic mechanism was reported to be based on the change of the wettability on the implant surface, reducing adhesion of molecules and cells to the coating through charge or hydrophobic interactions [243,244]. Various techniques have been adopted to produce superhydrophobic materials, including chemical vapor deposition, fluorine-based polymer, and laser system among the others two steps strategies. On the other hand, other authors developed a one-step methodology to fabricate a superhydrophobic coating for the titanium implants using glow discharge plasma. The results demonstrated an improvement in corrosion resistance, good biocompatibility, antimicrobial ability, and a reduction of colonization by oral pathogens [245].

## 5. Latest Trends in Analytical Chemistry

**Figure 10:**
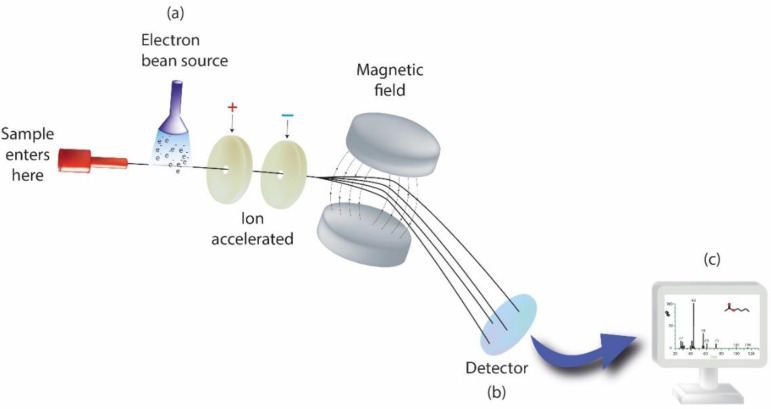
Schematic drawing of sample analysis by the use of a mass spectrometer. Briefly, the instrument consists of three components: (**a**) ion source, where gaseous ions are formed; (**b**) detector system, for detecting the ions. (**c**) Analyzer, for resolving the ions in accordance with their mass-to-charge ratio (m/z). The multiple ions generated from the injected sample are separated in function of m/z, and then recorded by considering their relative abundance.

The therapeutic impact of dental implants on human health has led to the need to develop reliable analytical methods as pillar for the evaluation of new biomaterial-based devices. Moreover, Singh et al. reported that “oral fluids (saliva) contains the biomarker and serum proteome components which enables the identification of both oral and systemic diseases” [246]. The quantification of markers’ concentration have had a very important role, due to the limitations of common methods for diagnosis and prognosis of peri-implantitis and oral issues [232,247,248]. In addition, the pharmacokinetics and pharmacodynamics properties of drug release from a biomaterial-based implant can be evaluated throughout high performance liquid chromatography coupled with mass spectrometry (Figure 10) or with an ultraviolet apparatus, considered the gold standard in this field [197,249,250,251]. In is reported, in the United States of Pharmacopeial Convention, that “the types of chromatography useful in qualitative and quantitative analysis employed in USP procedure are column, gas, paper, thin-layer (including high-performance thin-layer chromatography), and pressurized liquid chromatography (commonly called high-pressure or high performance liquid chromatography)”, where USP stands for United States Pharmacopeia [252].

The determination of some antibiotics has been extensively investigated. Thus, a gentamycin release profile has been carried out via high-performance liquid chromatography (HPLC), equipped with a reverse phase column, and coupled to an ion trap mass spectrometer with an electrospray ionizer. A mass spectrometry (MS) analysis confirmed the presence of three ions for the three components that form gentamycin (i.e., gentamicyn C1a, C2, and C1). The sum of these three most abundant ions was used for the calculation of gentamicin concentration [253]. Tobramycin quantification has represented a challenge in analytical chemistry due to its physico-chemical properties (i.e., basicity, hydrophilicity and lack of a UV absorbing chromophore) [254]. Sorensen et al. [112] evaluated the level of tobramycin incorporated and released from pins in PBS at 37 °C and analyzed by HPLC according to the British Pharmacopoeia and Fabre et al. [255], by the use of precolumn derivatization of the aminoglycoside antibiotic. On the other hand, tetracycline concentration released by nanofibers for dental coatings was easily determined by HPLC equipped with UV-Vis detector [167]. Moreover, in this last section, a brief panorama of novel analytical trends related to the use of biomaterials in dental implantology is presented, including the latest concept on the correlation between the etiogenesis of peri-implantitis and the degradation of the implant surface.

### 5.1. OMICS in Dental Implantology

Mass spectrometry has been considered the most comprehensive strategy in “OMICS” [256], which refers to scientific fields ending with –omics such as genomics, transcriptomics, proteomics, or metabolomics [257]. Besides, the development of analytical methods to characterize polymer sequences has represented a challenge in current polymer science, due to efforts to tailor the properties of biomaterials for medical purposes, among others [256]. In addition, OMICS technologies have gained momentum in uncovering molecules and signaling pathways related to bone formation and osseointegration, in order to provide personalized treatment in dentistry and implantology [258]. The bioconjugate BMP2-(PEO-HA)2, composed of a dendron with two monodisperse poly(ethylene oxide) branches functionalized with a HA binding peptide, and a focal point substituted with a bone-growth stimulating peptide (BMP2), was successfully characterized by MS methods. With this aim, the authors took advantages of various techniques including matrix-assisted laser desorption ionization (MALDI), electrospray ionization (ESI), tandem mass spectrometry (MS/MS), and ion mobility mass spectrometry (IM-MS) [259].

### 5.2. Detemination of Biomarkers for the Evaluation of Biomaterial Effect and Clinic Outcomes

The National Institutes of Health Biomarkers Definitions Working Group defined biomarkers as “a characteristic that is objectively measured and evaluated as an indicator of normal biological processes, pathogenic processes, or pharmacologic responses to a therapeutic intervention” [260]. The proper evaluation of biomarkers in an oral cavity can represent a useful tool for evaluating the activity-therapeutic effect of new biomaterial used for dental-implant coating. 

It was reported that various biomarkers were explored for the assessment of bone regeneration and healing around biomaterials. Many biomaterials were tested (e.g., titanium dental implants with surface modifications, and scaffolds loaded with drugs, osteogenic cells, or biological factors, etc.) in different trials. Bone regeneration, resulting in a biomarkers increase was reported in all these studies [261]. In particular, the rate of success of titanium dental implants can be monitored by the assessment of markers of osseointegration such as OCN (osteocalcin) and COL-1 (collagen type I) in the post-implant placement period [262,263,264]. In another study where the titanium dental implant was coated with a fluoride-material, the expression of OCN, RUNX-2 (Runt-related transcription factor 2), and COL-1 was associated with the positive effect of fluoride upon bone formation [265]. Likewise, a simvastatin coating proposed for in vivo trial, demonstrated the promotion of angiogenesis and osseointegration with the increased expression of VEGF and ALP markers [261,264].

With a similar aim, Kumar et al. evaluated the response of peri-implant connective tissue to titanium and zirconia abutments via the evaluation of MMP8 assayed by ELISA [266]. In fact, during the last decade the number of studies examining biomarkers in oral fluids as diagnostic tool for periodontal disease has represented a new trend. Since the 1920s, there have been many changes in the classification of periodontal diseases in order to obtain a proper diagnosis for the further treatment [8]. Saliva, gingival crevicular fluid, peri-implant sulcular fluid, and mouth rinse remnant have been considered non-invasive and biomarkers and high-content sources to reflect periodontal health. Furthermore, MMP8 (matrix metalloproteinase), MMP9, MMP13, MMP14, IL1β (interleukin), IL10, TIMP1 (metallopeptidase inhibitor), elastase, cathepsin G, cathepsin B, trypsin-like enzyme, sialidase, VEGF, RANKL (receptor activator of nuclear factor kappa beta), OPG (osteoprotegerin), TGF-β1 (tumor growth factor), nicotine, cotinine, cystatin C, MPO (myeloperoxidase), PAF (platelet-activating factor), and lactoferrin represent the common markers that researchers assayed over the last decade. On the other hand, ELISA, immunofluorometric assay (IFMA), colorimetric assay and chromatography-tandem MS have been selected for their determination, as previously reported by Gul et al. [247].

### 5.3. Analytical Methods for the Characterization of Micro- and Nanosized Implant-Related Particles and Metals in Inflamed Peri-Implants Ttissues

An emerging concept about etiopathogenesis of peri-implantitis has explored the degradation of the implant surface with the consequent particle release as an inflammation catalyst mechanism [267]. This phenomenon has been deeply studied for orthopedic implants, whereby the deterioration of metal biomaterials was demonstrated to accumulate exogenous particles in the peri-implant milieu [268]. Likewise, some authors reported that in dental devices, damage to the surface can be caused by both surface instrumentation and dynamic interactions in the implant–abutment interface; however, for titanium devices this can happen, independently from wear corrosion, following a no pre-clinical studied pathway [267,269]. In addition, differences among cell compositions were noticed in case of inflammation in peri-implantitis and periodontitis, with higher macrophage polarization in the first case [270,271]. However, other histological studies, conducted in presence of inflammation around titanium and ceramic implants, demonstrated that implantitis issues depend on a patient-level rather than a material level [272].

Taking into account that implant-surface degradation may result in the release of titanium ions, as well as particles, which leads to peri-implant inflammation and clinical failure, and it was hypothesized that release can occur in cases for which titanium implants are exposed to corrosion. This study demonstrated, for the first time, via synchrotron X-ray fluorescence mapping, a scattered and heterogeneous distribution of titanium in inflamed tissues [273]. Besides, the release of particles from Ti and ZrO_2_ implants has been observed in pigs after 12 weeks of implant placement [274]. Thus, to explore size, distribution, and the chemical speciation of substances that can be released from dental implants, Nelson et al. proposed a synchrotron-based characterization of micro- and nanosized implant-related particles arising from Ti and ZrO_2_ dental implants in patients with peri-implantitis. For this purpose, synchrotron μ-XRF, nano-XRF and μ-XANES were employed as an analytical tool. They also tried to explain the mechanism behind particle release from ceramic implants as a consequence of stress that induces the local transformation of zirconia from a tetragonal to a monoclinic crystalline phase, susceptible to microlesions. The study concluded by reporting on the presence of Ti particles, with variable speciation, in all tissue sections implanted with Ti devices, as well as ceramic products were identified in five out of eight tissue samples around ceramic implants [267].

**Figure 11:**
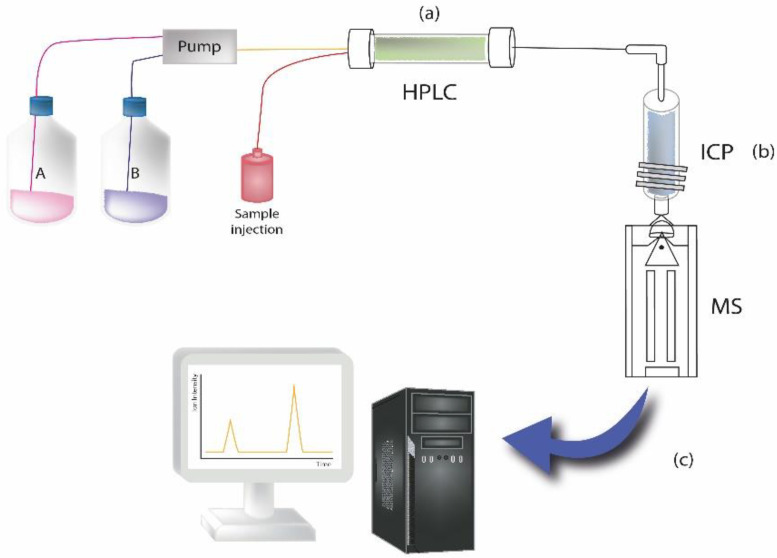
The illustration represents an example of sample analysis carried out by HPLC coupled to inductively coupled plasma mass spectrometry to measure elements at trace levels in samples. Briefly, the instrument consists of the following components: (**a**) HPLC system, where the chromatographic separation of the sample takes place; (**b**) ICP system, where plasma is ionized by inductively heating the gas (i.e., Argon) with an electromagnetic coil; it represents the ionization source. (**c**) MS detector system, for detecting the formed ions.

Because of the possibility of degradation of the implant surface within metals release, high performance liquid chromatography coupled with inductively coupled plasma mass spectrometry (HPLC-ICP-MS) was used for the study of distribution and chemical speciation of metals [275,276]. Balcaen et al. developed a reliable method for the determination of trace levels (limit of detection down to 3 ng L^−1^) of titanium in human serum, based on the use of ICP-MS/MS. In fact, it has been reported that the presence of implants in the human body can result in the high presence of titanium in serum [277]. Other researchers provided HPLC-ICP-MS (Figure 11) as a method for the estimation of total chromium and Cr(III) and Cr(VI) species released from metal implants into whole blood and joint effusion. The results showed higher chromium levels in joint effusion samples obtained from implanted patients [275].

Laser ablation-inductively-coupled-plasma mass spectrometry (LA-ICP-MS) was employed as an analytical procedure for the determination of elements derived from titanium implants and physiological elements in soft tissues. Results showed a quantitative mapping of Ti and Al released from dental implant and Mg, Ca, Fe, Zn, Cu, Mn as physiological elements in oral mucosa. Moreover, authors were able to obtain two-dimensional maps of distribution of elements in tested samples which confirmed the release of Ti and Al derived from implants [278].

## 6. Conclusions and Future Perspectives

These results broaden the understanding of the development of new materials within the area of dental implantology, suggesting that new multidisciplinary research is needed to improve the biological, physical, and chemical performance of these prostheses. An examination from an engineering perspective shows that various strategies, including the correct selection of both biomaterial composition and surface/geometry of the implant, confirm that they play a key role in achieving good compatibility and promote osseointegration after implantation surgery. Additionally, the implementation of organic- or inorganic-based coatings to promote not only osseointegration but also efficient pharmacological devices that could act as excellent therapeutic agents, including a polymer matrix that allows smart drug delivery in situ, emphasizes the importance of developing future method by which to cope with the 5–11% of dental implants that fail.

Beyond the prospect of exploring accurate analytical methods to evaluate the efficiency of these devices, MS appears as one of the most adequate, since it covers the molecule composition to unravel the mechanistic pathways of complex reactions such as bone formation. Moreover, clinical studies are being employed to manage the implication of biomarkers related to inflammation, osteointegration, or healing procedures via ELISA to measure and evaluate the activity and therapeutic effect of novel biomaterials, biological processes, and pathogenic responses.

This compilation provides a jumping-off point for the use of new biomaterial design for dental implantology and will bring research attention to include alternative methods for a better and more complete understanding of processes from an interdisciplinary point of view. Furthermore, not only will these advancements pave the way for a brighter future for patients with weak or sparse bone who will probably enjoy a better prognosis for successful implants, but other areas such as nanodentistry or nanotechnology are also expected to impact not only diagnosis, but also materials, to support the idea that better is not enough; the best is yet to come.

## Data Availability

The data presented in this study are available on request from the corresponding author.
